# Illegal markets for cigarettes and e-cigarettes

**DOI:** 10.1073/pnas.2512084122

**Published:** 2026-08-10

**Authors:** Donald Kenkel, Grace Phillips

**Affiliations:** ^a^ Department of Economics, Jeb E. Brooks School of Public Policy, Cornell University, Ithaca, NY 14853; ^b^ Department of Economics, University of Memphis, Memphis, TN 38152

**Keywords:** illegal markets, cigarettes, e-cigarettes

## Abstract

This paper discusses the long-standing illegal markets for cigarettes and the more recently emerged illegal markets for e-cigarettes. We identify three distinctive market fundamentals that help explain many features of the illegal markets. First, in contrast to some illegal goods, many countries have sizable legal markets for cigarettes and e-cigarettes alongside the illegal markets. In 2024, tobacco products generated almost $1 trillion in revenues; e-cigarettes generated $26 billion dollars. It is estimated that illegal markets account for about 10 percent of global cigarette consumption. Second, the illegal markets have arisen to evade high excise taxes that increase prices and regulations that restrict the availability of desirable features of legal products. The taxes and regulations have been adopted as public health measures with the goal of reducing tobacco consumption; however, these same policies drive the formation of illegal markets. Third, demand-side linkages mean that taxation, regulatory, and enforcement policies targeted at legal and illegal markets for cigarettes will affect legal and illegal markets for e-cigarettes, and vice versa. The connection between the markets means regulatory policy for one product can drive consumers both to the illegal market for the product and to the legal and illegal market for the other product.

In this paper, we discuss the long-standing illegal markets for combustible tobacco products and the more recently emerged illegal markets for consumer products that provide nicotine without the combustion of tobacco (Box 1).^*^ Our discussion will focus on cigarettes and e-cigarettes, which are the most popular examples of each product type and account for the largest shares of the illegal markets.^†^ We identify three distinctive market fundamentals that help explain many features of the illegal markets. First, in contrast to some illegal goods, many countries have sizable legal markets for cigarettes and e-cigarettes alongside the illegal markets. In 2024, tobacco products generated almost $1 trillion in revenues, mainly from cigarette sales; e-cigarettes generated $26 billion dollars (1–3). While the share of the illegal markets varies by country, it is estimated that illegal markets account for about 10 percent of global cigarette consumption (4). Second, the illegal markets have arisen to evade high excise taxes that increase prices and regulations that restrict the availability of desirable features of legal products. Third, demand-side linkages mean that taxation, regulatory, and enforcement policies targeted at legal and illegal markets for cigarettes will affect legal and illegal markets for e-cigarettes, and vice versa.

Box 1. Tobacco and Consumer Nicotine Products.

**Table:** 

Combustible tobacco products	Consumer products that provide nicotine without combustion of tobacco
Cigarettes	Disposable “cig-alike” e-cigarettes
Cigars	Cartridge- or pod-based e-cigarettes
Small cigars/cigarillos	Large Capacity disposable e-cigarettes
Pipe tobacco	Open refillable systems and e-liquids
Roll-your-own tobacco	Heated tobacco products
	Nicotine pouches
	Oral tobacco products (snuff, chewing tobacco, and snus)

## 1. Origins and Motivations

The long-standing illegal markets for cigarettes stem from the equally long-standing public policy of imposing excise taxes on cigarettes. Separate from general sales taxes and value-added taxes, as of 2022, 168 countries imposed specific excise taxes on cigarettes (5). In high-tax countries, illegal trade in cigarettes is a profitable form of tax evasion. Illegal nontaxed cigarettes can be sold at much lower prices, undercutting the prices of legal cigarettes.

In addition to excise taxes, the regulations that some countries impose on cigarettes may further increase demand in illegal markets. One newer policy trend is to prohibit the addition of menthol flavoring to cigarettes. Menthol cigarettes are currently prohibited in Canada, Ethiopia, Great Britain, the European Union, and in two states and 190 localities in the United States (6, 7). The typical legal approach is to prohibit the sale of menthol cigarettes, while not criminalizing consumer possession or use.

In contrast to illegal cigarette markets, e-cigarettes are sold in illegal markets mainly to evade prohibitions or regulations. Tax evasion currently plays a smaller role because e-cigarette taxes are generally lower or nonexistent, but this might change if high e-cigarette excise taxes or import tariffs become more common. As of 2022, 34 countries prohibit the sale of e-cigarettes. E-cigarettes are legal but regulated in 87 countries, while 74 countries do not regulate e-cigarettes (5). Some e-cigarette regulations, like age restrictions and bans on indoor vaping, parallel cigarette regulations. Another e-cigarette policy trend is to restrict the availability of desirable features such as fruit and sweet flavors. In general, prohibitions and flavor bans apply both to closed system e-cigarettes and to the e-liquids used in open refillable systems. In 2021, Australia adopted a novel approach to e-cigarette regulation and required a physician’s prescription, which in 2024 was replaced with the requirement that e-cigarettes can only be legally sold in pharmacies.

The taxes and regulations that drive illegal cigarette markets and illegal e-cigarette markets are seen as policy tools to improve public health; excise taxes also generate revenues. The World Health Organization considers excise taxes to be the most effective tobacco control policies (5). Cigarette tax revenues tend to account for a large share of public sector budgets in low-income countries that face difficulty enforcing and collecting revenues from other taxes. In high-income countries, where excise taxes are typically used as public health policy tools, cigarettes are generally more heavily taxed than in low- and middle-income countries, both in absolute terms and relative to the price of cigarettes. Policies that regulate cigarette attributes also reflect the goal to improve public health, even at the cost of losing excise tax revenues. For example, to the extent menthol bans achieve their intended consequence to reduce smoking, they will also reduce cigarette excise tax revenues. The political economy of the regulation of cigarette attributes thus differs from the political economy of cigarette taxation. Higher cigarette taxes can have wider appeal, both to public health advocates and to advocates of balanced public sector budgets. Regulation of cigarette attributes poses tradeoffs between public health and tax revenues.

The prohibitions and regulations that drive illegal e-cigarette markets are also seen as policies to improve public health, especially by preventing teen use. For example, the US de facto ban on e-cigarette flavors other than tobacco and menthol reflects Food and Drug Administration (FDA) findings that there is not sufficient evidence of additional benefits for adult smoking cessation to offset the risks to teens, who disproportionately prefer flavored e-cigarettes (8). Similarly, Australia’s policy to only allow e-cigarette sales behind the counter in pharmacies is intended to make e-cigarettes available to adults for smoking cessation while preventing teen access.

Because the common goal of cigarette and e-cigarette policies is to improve public health, the stringency of the policies often tends to be correlated across jurisdictions. For example, Australia heavily taxes cigarettes and strictly regulates the sale of e-cigarettes. However, other countries recognize a tradeoff between e-cigarette regulations to discourage teen use versus making e-cigarettes available for adult smoking cessation. Because the health risks from smoking stem from the combustion of tobacco, e-cigarettes are a less risky source of nicotine (9). Nicotine is addictive but is not linked to the major causes of smoking-related deaths (10). In the United States, e-cigarettes are regulated by the FDA. While no tobacco product is risk-free, the FDA recognizes that tobacco products exist on a “continuum of risk,” with combusted products like cigarettes as the most harmful and noncombusted products like e-cigarettes as lower-risk alternatives (11). Recognizing this tradeoff, the FDA has authorized some tobacco- and menthol-flavored e-cigarettes as appropriate for the protection of public health because of their potential to help adult smokers quit (12). The United Kingdom goes further and actively promotes e-cigarettes for smoking cessation.

## 2. Scale of Markets and Harms

When considering the scale of illegal markets for cigarettes and e-cigarettes, it is useful to characterize types of illegal trade by where in the supply chain the illegality occurs. Products sold in illegal markets may have been manufactured illegally, or the products may have been manufactured legally but distributed illegally to evade taxes or regulations. Because regulations vary across countries, products such as flavored e-cigarettes are legal in some countries but become illegal when distributed and sold in other countries. Tax evasion and regulatory noncompliance are always illegal, but the seriousness of the offenses varies. The illegal actions are typically taken by the distributors and vendors, not the consumers. In fact, in many jurisdictions, consumers can legally avoid taxes or regulations on products purchased legally in another jurisdiction, as long as the purchases are for personal use. In this section, we review estimates of the overall scale of illegal markets for cigarettes and e-cigarettes and descriptive evidence on where in the supply chain the illegality occurs and on the severity of the offenses.

As with all illegal markets, there are no authoritative measures of the size of illegal markets for cigarettes and e-cigarettes. However, the existence of legal markets provides leverage for measurement. A National Research Council study discusses multiple methods that have been used to measure the size of illegal cigarette markets (13). One set of studies infers the size of the illegal market from the trade gap, i.e., legally manufactured cigarettes that are labeled as intended for export but are diverted into illegal markets during transit. Another set of studies compares self-reported consumption of cigarettes to administrative data on tax-paid cigarette sales. Some population surveys directly ask smokers about illegal purchases. Another approach to measure the size of illegal cigarette markets uses data on whether discarded cigarette packs have the appropriate tax stamps.

The World Bank and the World Health Organization cite consensus estimates that illegal markets account for about 10 percent of global cigarette consumption (4, 5). Illegal markets’ share of global cigarette consumption appears to have been fairly stable for decades; reviews of studies from around 1995, 2007, and 2015 put the share at 8.5 percent, 12 percent, and 11 percent, respectively (14–16). The size of illegal cigarette markets varies widely across countries. Using Europe as an example, in 2010, the World Health Organization’s Framework Convention on Tobacco Control estimated that illegal cigarettes comprised, on average, 10 percent of the EU countries’ cigarette market. In Latvia, illegal cigarettes were estimated to comprise 41 percent of the market while in the Ukraine, illegal cigarettes were estimated to be only 1.5 percent of the national market (17).

A large body of research explores illegal cigarette markets in the US. The National Research Council study concludes that illicit cigarette sales accounted for between 8.5 percent and 21 percent of the total US cigarette market, with wide variation across states (13). The National Research Council’s estimate of “illicit” cigarettes includes illegal tax evasion and legal tax avoidance. Another study reports results from collecting discarded packs in five large US cities (18). Based on assumptions based on the origins of packs without the required local tax stamps, the study concludes that between 30.5 percent and 42 percent of discarded packs were supplied by illegal wholesalers. Although these estimates are for the narrower category of illegal tax evasion, they are larger than the National Research Council’s national estimate that includes legal tax avoidance. Two factors help explain why. First, four of the five cities in the study are in states with high taxes. Second, the discarded pack method used tends to result in higher estimates of illegal market shares.

Less is known about the size of illegal e-cigarette markets both globally and in specific countries. In countries where e-cigarettes are banned, self-reported consumption provides a lower-bound estimate of the size of the illegal market. For example, when nicotine e-cigarettes were only legal by prescription, an impact analysis of new legislation to improve compliance concluded that between 3 and 12 percent of the Australian market for nicotine e-cigarettes was legally supplied to vapers with a prescription (19); an online survey found that between 3 and 24 percent of current vapers were compliant with the prescription requirement (20). Put differently, the compliance rates imply that illegal sales accounted for between 76 and 97 percent of nicotine e-cigarette use in Australia.

It is more difficult to develop measures of the size of the illegal e-cigarette market in countries where e-cigarettes are regulated but not banned. Self-reports or sales data for e-cigarettes with the regulated attribute, e.g., fruit and sweet flavors, again can provide a lower-bound estimate of the size of the illegal market. Through July 2025, the US FDA has authorized 39 tobacco-flavored and four menthol-flavored e-cigarettes but has not authorized any e-cigarettes with fruit and sweet flavors. Despite the lack of authorized flavored products, more than half of the e-cigarettes sold in 2024 and 2025 came from illegal nontobacco and nonmenthol flavored e-cigarettes (21). Taken together, FDA-authorized e-cigarettes are calculated to account for 14 percent of e-cigarette sales in convenience stores in 2024 (22). The remaining 86 percent of e-cigarette sales are illegal but differ in the seriousness of the offense. Some manufacturers have submitted applications to the FDA, but their applications are still under review or in litigation; the FDA tends to use its enforcement discretion in these cases. The distribution and sale of products that are not under FDA review and products that have been the subject of FDA enforcement actions are more serious offenses.

Illegal cigarette and e-cigarette markets may give rise to the same harms created by other illegal markets. One concern is that illegal markets lead to violence. The review by the National Research Council notes that the people involved in modern European illegal cigarette markets generally do not have serious criminal records, and the markets do not seem to be associated with violence (13). However, if illegal cigarette and e-cigarette markets grow, the markets may become more violent. In a recent example from Australia, an investigation of tobacco smuggling identified links to a major criminal syndicate involved in money laundering and violence (23). A somewhat different concern in the US context is that illegal cigarette markets may lead to violent interactions between police and the public. For example, the high market share (85 percent) of menthol cigarettes among US Black smokers raises unique issues for racial disparities and racial justice. The racial justice concerns about unequal enforcement of a menthol prohibition are especially salient in light of the death of Eric Garner who was killed by police in an attempt to arrest him for selling illegal single cigarettes (24). Another broad concern about illegal markets is that they are a source of profits for organized crime groups and other violent groups.

The harms of illegal markets for cigarettes are mostly associated with the health harms of smoking, whether or not cigarettes are illegal. In addition, as with other substances sold in illegal markets, due to lack of regulation illegal cigarettes and e-cigarettes might be more likely to be contaminated. In 2019, an outbreak of lung injuries in the United States was initially found to be associated with vaping e-cigarettes. Further Centers for Disease Control investigation determined that the outbreak was linked to illegal vaping products that contained tetrahydrocannibinol, the psychoactive component of cannabis, and a contaminant. The outbreak was not due to the commercially manufactured nicotine-containing e-cigarettes discussed in this paper. However, illegally supplied open refillable e-cigarettes systems pose a risk of contamination. Unregulated manufacturing processes also pose a risk. Finally, illegal markets may tend to be sold disproportionately to high-risk individuals, e.g., adolescents.

## 3. Structure and Nature of Illegal Markets, Supply Chains, and Criminal Enterprises

### 3.1. Market Structure.

Illegal cigarettes can be described as falling into three categories: contraband, so-called cheap or illicit whites, and counterfeits. Contraband cigarettes are legally manufactured branded cigarettes that are smuggled across borders to avoid duties and taxes. Cheap whites are, as suggested by the term, inexpensive cigarettes with unique brand names or no brand names. Cheap whites can be legally or illegally manufactured. Counterfeits are illegally manufactured cigarettes sold under brand names without consent of the holder of the trademark.

The expert consensus is that contraband cigarettes and cheap whites make up most of the global illegal market in cigarettes. Based on reported seizures of illegal products and on industry reports, contraband cigarettes are estimated to account for around 60 to 70 percent of the illegal market, cheap whites account for around 20 to 30 percent, and counterfeits account for less than 10 percent (25). Notably, in contrast to Europe and other parts of the world, cheap whites do not appear to be popular in US illegal markets, which might be due to higher-income consumers favoring higher quality, brand-name cigarettes. Illegal e-cigarettes are typically contraband products that are legally manufactured and then smuggled across borders to avoid regulations or bans. There does not appear to be a close e-cigarette analogue to cheap whites. Counterfeit e-cigarettes also do not appear to be common.

Because most cigarettes and e-cigarettes sold in illegal markets are manufactured legally, the structure of the legal market is the starting point to understand the structure of the illegal market. In many countries, the cigarette industry is a tight oligopoly with a small number of major manufacturers, each of which has a relatively large share of the market. The market for cheap whites is more competitive, with smaller manufacturers located in various countries. Due to brand loyalty and quality differences, cheap whites are imperfect substitutes, which allows the major manufacturers to maintain their market power in the legal market.

To some extent, the legal cigarette market structure carries over into the illegal markets. Contraband products from the major manufacturers and cheap whites compete but mainly in different market segments and price points. The market power of the major manufacturers may tend to increase the wholesale prices of their cigarettes before they are diverted into illegal markets. The prices of untaxed cheap whites are lower than the prices of untaxed contraband cigarettes from the major manufacturers.

The extent to which the legal market structure carries through to illegal markets depends on the role the major cigarette manufacturers play in the illegal market and afterward. Because any direct roles would involve illegal acts, their roles are difficult to determine. In the late 1990s, investigations by the European Union and lawsuits documented illegal acts committed by major manufacturers and led to legal agreements to curb their involvement with smuggling (25). Based on this history, some observers suspect the major manufacturers continue to play active roles in the illegal market; or “[a]t best, evidence indicates that tobacco companies are failing to control their supply chain … in the knowledge their products will end up on the illicit market” (25). Manufacturers of cheap whites may play a more active role; one study notes that cheap whites “appear to be purposefully produced for the black market.” (26) Another study reports that cheap whites are often manufactured in “free zones” that countries designate for less restrictive regulation (27).

The structure of the legal e-cigarette market is also the starting point to understand the structure of the illegal markets for those products. Interestingly, major manufacturers of cigarettes are also major players in the manufacture and distribution of legal e-cigarettes in the United States. In the study of convenience store sales data, the brand of e-cigarettes with the highest market share (32 percent in 2024) is made by Reynolds American (22). The role of large cigarette manufacturers in the legal market for e-cigarettes might reflect the barrier to entry created by the costly FDA marketing authorization process.

To date, in the United States, the FDA marketing authorization process for e-cigarettes has resulted in a highly concentrated legal market and much more competitive illegal markets. Several of the leading brands in the convenience store data are made by Chinese firms that specialize in e-cigarettes (22). The brands made by Chinese firms are especially popular among teen vapers in the United States. In the 2024 National Youth Tobacco Survey, among current vapers, the three most commonly reported brands are made by Chinese firms (28). The brands made by the Chinese firms have not received FDA marketing authorization and are hence illegal, although they may be sold by otherwise legitimate retailers.

Patterns in the data also show that US illegal markets for e-cigarettes are dynamic. For example, in the convenience store data between 2022 and 2024, the two leading Chinese-made e-cigarette brands increased their combined market share from 4 percent to 20 percent (22). In the National Youth Tobacco Survey data, although the same brand was the most reported brand in 2023 and 2024, the fraction of youth reporting that brand dropped by 21 percentage points in the 2024 survey (28, 29). The study of convenience store data also finds that some brands with small market shares at the national level emerge as leading brands in some regions. The authors suggest that some brands’ higher market shares in certain regions “is likely due in part to the supply chain and proximity to the headquarters of the manufacturer or distributor.” (22)

In addition to considering the structure of illegal markets for cigarettes and e-cigarettes separately, it is sometimes useful to analyze them together as forming a single market for consumer products that deliver nicotine. Because nicotine is the addictive component of tobacco smoke, many smokers use e-cigarettes to cut down or quit smoking. A systematic review of evidence from randomized clinical trials concludes that there is high certainty evidence that use of e-cigarettes increases quit rates compared to pharmaceutical nicotine replacement therapies (30). A study using observational data from the 2022 US National Health Interview Survey finds that of the estimated 2.9 million adult smokers who had quit in the past year, 26 percent used e-cigarettes alone to quit smoking and 41 percent used e-cigarettes in combination with some other method (31). In industrial organization and antitrust economic analysis of market structure, a central question when defining a market is the extent to which the products are economic substitutes. A growing body of economic research provides empirical evidence that cigarettes and e-cigarettes are economic substitutes (32–34). Evidence that cigarettes and e-cigarettes are close substitutes means that taxation, regulatory, and enforcement policies targeted at illegal markets for cigarettes will affect illegal markets for e-cigarettes, and vice versa. Analyzing either illegal market separately runs the risk of missing important unintended consequences, such as the effects of e-cigarette flavor bans to increase cigarette demand.

### 3.2. Supply Chain and Criminal Enterprises.

We now turn to describing the supply chains of wholesalers and retailers of illegal cigarettes and e-cigarettes and the role of criminal enterprises. We use the term wholesalers to include the people and organizations that smuggle or otherwise divert legally manufactured products into the illegal supply chain, usually to evade excise taxes or regulations.

The key source of profits to illegal cigarette wholesalers is tax evasion and arbitrage of cross-border price differentials due to taxes. Globally in 2022, on average, excise taxes accounted for almost 50 percent of the retail price of cigarettes, and there is substantial variation around that average. In 22 countries, taxes accounted for less than 25 percent of the retail price, while in 23 countries, taxes accounted for 75 percent or more of the retail price (5). Smuggling untaxed or low-taxed contraband cigarettes into high-tax jurisdictions is a good source of profit for the illegal wholesaler. The smuggled cigarettes can be sold at a price low enough to undercut legal taxed cigarettes but high enough to cover the costs of smuggling, including a risk premium to compensate the illegal wholesaler for the possibility of apprehension and punishment. An econometric study of most countries in the European Union estimates that every 1 Euro increase in tax per pack increases the illegal market share by 5 to 12 percentage points (35). In a sample of 12 cities across the world, another study finds that higher cigarette taxes are associated with greater availability of untaxed cheap whites (36). Within the United States where cigarette taxes vary widely across states, a line of research finds that higher taxes lead to cross-border purchases (37–39). Because e-cigarette taxes are mainly low or nonexistent, the key source of profits for illegal e-cigarette is different—simply the ability to have access to demand in large markets, such as the demand in the United States for illegal flavored e-cigarettes.

Some illegal cigarette market wholesalers are much different than their legal market counterparts. A distinctive feature is the very wide range of organizations involved in cigarette smuggling, even within one country. For example, in illegal cigarette markets in Italy, smuggling operations range from large-scale operations that use container shipments hidden in other goods to individuals hiding contraband cigarettes in their private luggage (40). The wide range in the scale of the illegal enterprises means that cigarettes smuggled by the large-scale smuggling operations will tend to account for a high share of illegal market supply. To illustrate, in the World Customs Organization data for 2022, the 100 largest seizures accounted for 80 percent of all cigarettes seized. Seizures from vessels including container shipments accounted for 45 percent of cigarettes seized; seizures from air travelers’ private luggage accounted for less than 1 percent (41).

Some aspects of the supply chain for illegal cigarettes appear to have changed over time in many countries. For example, in the 1950s to 2000s, Italian organized crime groups imported large numbers of contraband branded cigarettes into Italy. From the 2000s onward, Italian organized crime groups entered into joint ventures with criminal organizations from Eastern Europe and China (40). Similarly, the New York Cosa Nostra was involved in illegal cigarette markets in the 1960s and 1970s. However, the US illegal markets apparently fragmented since the 1970s (26). It is not clear the extent to which organized smuggling of cigarettes is currently undertaken by criminal enterprises involved in other illegal activities, or by enterprises that specialize in cigarette smuggling. In the recent seizures of smuggled cigarettes and e-cigarettes in Australia, the authorities identified a major criminal syndicate that is also involved in money laundering (23).

Most illegal e-cigarettes appear to be manufactured legally and then diverted into the illegal supply chain. At least some of the wholesale/smuggling operations are large scale. For example, the defendants to a lawsuit filed in February 2025 include four corporations that import and/or distribute flavored disposable e-cigarettes, which were almost all made by Chinese manufacturers. Based on court documents, the four corporations provide e-cigarettes to tens of thousands of retailers, employ hundreds of employees, and have hundreds of million dollars in annual revenues (42).

The final link in the illegal e-cigarette and cigarette market supply chain—retailers—appears to be different for cigarettes and e-cigarettes. The illegal cigarette retail market is again distinguished by the variety of organizations. Qualitative ethnographic researchers have collected in-depth data on attitudes toward illegal cigarette markets in small samples of subjects. One study reports the results of focus groups conducted in May 2003 (shortly after a cigarette tax increase) with 104 residents of Harlem, a predominantly Black low-income community in New York City (43). Focus group participants used the term “$5 man” to describe a highly visible network of illegal dealers who sold cigarettes in public places such as street corners and near subway entrances. However, subsequent ethnographic research in the South Bronx neighborhood of New York City describes consumers favoring under-the-counter purchases from retailers like bodegas over purchases from illegal dealers on the street (44). By one recent estimate, there are about 8,000 unlicensed illegal smoke shops selling tobacco and cannabis products in New York City (45); this is about twice as many as the approximately 4,000 active tobacco retail dealer licenses and licensed cannabis dispensaries (46). Open street markets operate in some Italian cities, but also sales from private apartments (40). A distinctive feature of the retail sector is that some retailers sell legal cigarettes over-the-counter and illegal cigarettes under-the-counter or even more openly.

Perhaps even more often than in illegal cigarette retail markets, illegal e-cigarettes are frequently sold openly by licensed retailers who otherwise operate legally. The US convenience store data—which shows that in 2024, 86 percent of e-cigarette sales were for illegal products unauthorized by the FDA—were from Nielsen’s Convenience Track system (22). The Nielsen sales estimates are representative of the convenience store industry. The data come from in-store barcode readers in major chain and independent convenience stores and from audits of convenience stores without barcode readers. While the Nielsen data might miss additional under-the-counter sales of illegal e-cigarettes, clearly a large volume of illegal e-cigarettes is being sold openly. In response to the concern that retailers simply do not know which products are authorized, some states have created e-cigarette registries that list the products that can be legally sold.

Like the retailers themselves, many e-cigarette consumers may be unaware that they are making illegal purchases. The changing landscape of Australian e-cigarette regulation left many Australian vapers unaware that their purchases were illegal. Until 2024, e-cigarettes without nicotine were legal in most places in Australia, and e-cigarette importers were not required to prove that their products did not contain nicotine (47). As a result, “importers have flooded Australia with [nicotine e-cigarettes] simply by not declaring the nicotine content present.” (48). After importation, state-level enforcement was also difficult because laboratory testing is required to determine whether an e-cigarette contains nicotine. As a result, nicotine e-cigarettes were widely sold in retail outlets. Between 2021 and 2024, vapers were required to have a prescription to legally purchase e-cigarettes. However, an online survey found that only 40 percent of current vapers in Australia were aware of the prescription requirement (21).

Consumers can also purchase e-cigarettes from online retailers. In the 2022 wave of the US Population and Tobacco Health Survey, four percent of current vapers report purchasing their e-cigarettes online.^‡^ National, state, and local e-cigarette taxes and regulations are harder to enforce with online retailers, so a high fraction of online sales may be illegal products. Once again, the definition of legality can be murky. For example, when Australia’s prescription requirement was in place, individuals with a prescription or permit could legally import a limited quantity of nicotine e-cigarettes for personal use as an unapproved therapeutic good (49). Online e-cigarette retailers were an important source for legal personal imports. However, some online retailers also provided lists of physicians who provided prescriptions of questionable legality.

## 4. Enforcement and Policy Approaches

The coexisting legal markets mean that most enforcement efforts target the supply side of illegal markets for cigarettes and e-cigarettes. Despite the common focus on supply chains, how countries target illegal supply is context specific. Differences in geography, regulations, and proximity to illegal suppliers result in unique local illegal supply chains for both cigarettes and e-cigarettes. Despite the similarities in enforcement against the two illegal markets, the longevity of the legal and illegal cigarette market is paradoxically the reason enforcement is more successful against illegal cigarettes.

Compared to cigarettes, e-cigarettes are a relatively recent market innovation; as such, the regulation of e-cigarettes is also relatively recent. In addition, e-cigarette regulations are highly varied across countries. Countries like Australia or the United States strictly regulate e-cigarettes, while the United Kingdom’s regulation of e-cigarettes is comparatively more lenient.^§^ Given both the recency and variability of regulations, the market for illegal e-cigarettes is highly country specific, limiting the possibility of a global approach to the issue. A key component of successful enforcement against illegal cigarettes is a unified global approach.

The World Health Organization has spearheaded efforts to coordinate a unified global response to illegal cigarettes. In 2018, the Protocol to Eliminate Illicit Trade in Tobacco Products (ITTP) was adopted by the World Health Organization’s (WHO’s) Framework Convention on Tobacco Control^¶^ creating a treaty focused on addressing illegal tobacco markets (4). A key focus of the ITTP is attempting to control and secure the illegal supply chains through a global track and trace system. A global track and trace system would allow any tobacco product to be tracked through every point in the distribution process. If an illegal tobacco product is found, it can then be traced back to the point when the illegal product was diverted from the legal market and into the illegal market. Such a system would help countries identify the key suppliers in the illegal market and efficiently and target their enforcement actions.

While a global track and trace system would be considered the gold standard for addressing illegal tobacco, countries have faced difficulty in the implementation of the system. In 2019, the European Union implemented a track and trace system, which some advocates criticized due to the system’s connection to the tobacco industry (50, 51). The concern about the EU’s track and trace system is not universal. The World Bank Group’s review of countries’ illicit tobacco policies recommended the EU’s track and trace system as a “scalable example of a system of tobacco traceability that is sufficiently flexible to be implemented both at the regional and the single-country scale” (4).

Notably, some middle- and low-income countries began implementing track and trace systems before the ITTP, as a strategy to combat the revenue losses from tax evasion (4). In 2010, Kenya used an electronic cargo tracking system to track the transit of domestically produced cigarettes designated for sale in other countries (52). It took another 5 y for Kenya to fully implement a track and trace system that allows for validation of cigarette legality at any point. There is suggestive evidence that Kenya’s system helped combat the illegal tobacco market and slightly increased excise tax revenues from cigarettes and cigars (4, 52).

When properly implemented, track and trace systems are an efficient way to target enforcement. However, track and trace systems must operate over a large geographic area to work properly. An evaluation of state-level track and trace systems in the United States demonstrates the limitations of these systems when they are not adopted at a large enough scale (53). In the United States, only three states, California, Massachusetts, and Michigan, use a track and trace system. Given that the majority of illegal tobacco in the United States is derived from tax evasion, the current track and trace systems fail to capture any products smuggled in from low-tax states. The authors argue that a federal-level system would address these issues. However, the issues apply to larger-scale track and trace systems as well. A country-level track and trace system when faced with a smuggled or otherwise illegal product, can only show that the product is illegal. A country-level system cannot identify an illegal product’s origin if it does not enter the country legally and the country of origin does not have a track and trace system.

On the individual level, countries’ enforcement against illicit tobacco products is less structured and more piecemeal. Australia, for example, has focused on stopping local tobacco production and increasing enforcement against smuggled tobacco. In 2015, Australia stopped all domestic tobacco production (4). As of 2022, there was no legal tobacco manufacturing occurring in Australia, and there were no licenses “to grow or deal in tobacco seeds, plant or leaf, either for commercial or personal use” (54). At the same time, the Australian Border Force (ABF) increasingly focused on stopping the flow of smuggled tobacco.

With the introduction of e-cigarettes to the illegal market for tobacco, ABF, in conjunction with the Therapeutic Goods Administration, the Australian Federal Police, and local authorities, expanded their responsibilities to include the smuggling of e-cigarettes. Unlike track and trace systems, AFB operations tend to focus on individual raids or criminal networks. These operations aim to reduce the distribution of illegal tobacco and e-cigarettes by disrupting established supply chains and criminal networks. In 2024, one such operation executed 39 warrants across Sydney, which resulted in the seizure of 5.6 million cigarettes, 404 kg of loose-leaf tobacco, and over 200,000 e-cigarettes (55).^#^

Unlike the illegal cigarette market, there is not a global initiative to address the illegal e- cigarette market. The WHO has only recently extended the purview of its public health work on the global tobacco epidemic to encompass e-cigarettes and similar nicotine delivery systems (5). Their illegal tobacco efforts do not yet address illegal e-cigarette markets.

Across the world, the enforcement against the illegal e-cigarette market has been piecemeal. Each country crafts its own approach based on its e-cigarette laws and citizens’ e-cigarette use. Given the significant variation in attitudes toward and regulations of e-cigarettes, and the resulting illegal markets, this section will focus on enforcement in Australia and the United States. In the United States, enforcement against e-cigarettes is even more disjointed, given the variation in state laws regarding e-cigarettes and the FDA’s current approach to product authorization.

Rather than outright banning all flavored e-cigarettes, the FDA product authorization approach has created a regulatory gray area. While all unauthorized e-cigarettes are illegal, the seriousness of the violations (and therefore, the severity of punishment) varies. There are e-cigarettes that are smuggled in and sold with no pretense of complying with the FDA authorization process. Some e-cigarettes that are imported while under the review process are at least partly complying but are still illegally sold. Then there are imported e-cigarettes that have been denied authorization yet are sold illegally in violation of FDA orders.

The complex legal status of e-cigarette products places a significant burden on enforcement agencies. Enforcement agencies must be aware of products on an individual level and know each product’s authorization status. Both the relative recency of these regulations and the complicated regulatory environment make it harder for the United States to effectively address these illegal markets, which has led to fragmented enforcement against e-cigarettes.

At the federal level, the FDA sends warning letters to brick-and-mortar retailers, online retailers, and e-cigarette manufacturers that continue to sell unauthorized e-cigarettes (5, 56–61). If the retailers do not stop selling unauthorized e-cigarettes, subsequent FDA action can include injunctions, seizures, and civil money penalties. The maximum penalty for a single violation is $21,348, with the potential for the FDA to seek added penalties for certain intentional violations.^||^ Out of over 1,500 warning letters issued (700 plus to manufacturers and 800 plus to retailers), the FDA has currently filed a civil money penalty against 89 manufacturers, 146 brick and mortar retailers, and 46 online retailers (62). Given the limited enforcement actions leveed to date, the deterrence effect appears muted. Other federal action includes the formation of a joint multijurisdictional task force aimed at addressing illegal e-cigarette markets. This task force resulted in hundreds of seizures by both the FDA and Customs and Border Protection, with each seizure including millions of unauthorized e-cigarettes (63–65). For context, in 2024, there were 286 million e-cigarettes sold in US convenience stores (22).

At the local level, states with stricter local laws are taking action against illegal e- cigarette distributors and manufacturers. As of May 2025, five states and four cities have filed suits against e-cigarette manufacturers and distributors (66). Additional states have begun sending warning letters or are initiating investigations into e-cigarette manufacturers and distributors as of 2025.

Several states and counties have enacted their own statutes banning flavored e-cigarettes, providing a direct path to enforcement (7). Local e-cigarette laws give local law enforcement the jurisdiction to address illegal e-cigarettes. In states and counties without local bans, the jurisdiction of local authorities is more nebulous. The sale of e-cigarettes without a license directly violates state laws, thus falling under the jurisdiction of local authorities. However, e-cigarettes that have not been FDA authorized sold by otherwise licensed vendors fall into a gray area for local enforcement.

As the US case study illustrates, it is especially difficult to use enforcement to address illegal e-cigarettes. Until recently, consumers had legal access to products that are now illegal and significant demand for e-cigarettes persists after regulations’ implementation. The prohibition of once legal products has a long history of creating illegal markets, and in many cases, the illegal markets have been more successful than enforcement efforts.

A more promising approach to limiting illegal cigarette and e-cigarette markets while improving public health might be to continue and expand antismoking efforts. The US and other high-income countries have had considerable success in reducing tobacco use. In the United States, the adult smoking rate has fallen from 42 percent in 1965 to 11.5 percent in 2021 (67). The most recent data also show very low rates of teen smoking and declining rates of teen vaping (68, 69). These promising trends in teen smoking and vaping could reduce concerns about the enforcement of e-cigarette regulations in the United States. However, smoking rates remain much higher in other middle- and low-income countries.

Disrupting illegal markets is difficult. Some antismoking policies show promise in reducing the demand for legal and illegal cigarettes, while other antismoking regulations produce the demand for illegal markets. Both illegal cigarette and e-cigarette markets are especially difficult to disrupt via enforcement actions due to their robust legal markets. Multinational and large-scale approaches as described in the Protocol to Eliminate Illicit Trade in Tobacco Products (ITTP) show promise but are costly and time intensive to implement. Like other illegal markets, the most successful enforcement and regulations are often multifaceted and require both a supply chain–focused approach in conjunction with targeted one-off actions directed at individual sellers or crime networks. Unfortunately, these approaches are often slow to implement and costly, especially as the supply side adapts to the new measures.

## 5. Market Developments and Innovation

The basic products, supply chains, and nature of the criminal enterprises involved in illegal markets for cigarettes have shown some changes over the past 25 y. Illegal markets for e-cigarettes have been even more dynamic, with major product innovations.

Product innovations in illegal e-cigarette markets build on a track record of innovation in the legal market. The first modern e-cigarettes were widely introduced less than 20 y ago. The early disposable e-cigarettes known as “cig-a-likes” did not deliver nicotine as effectively as combustible cigarettes, so they were not too popular among smokers. An innovative pod-based product was introduced in 2015. By using nicotine salts, the pod-based product provided users with nicotine at a speed closer to the uptake from combustible cigarettes. Flavored pod-based and cartridge-based e-cigarettes and flavored e-cigarette liquids used in open-tank systems quickly became popular not only among adult smokers but also among youth. The rapid growth in teen vaping spurred new regulations, which, starting around 2020, spurred the growth of illegal markets.

Since 2020, innovation in e-cigarettes sold in illegal markets include innovations in reaction to regulation. Because the FDA’s regulatory authority over e-cigarettes was established by the Tobacco Control Act, instead of using nicotine derived from tobacco, to avoid regulations some e-cigarette manufacturers switched to synthetic nicotine. In 2021, the Act was amended to define synthetic nicotine as a tobacco product for the purposes of the Act. In a further response, instead of using either nicotine derived from tobacco or synthetic nicotine, at least one manufacturer launched products that use metanine, which does not fall under the Tobacco Control Act’s regulatory powers. Metanine is a nicotine-like chemical and is claimed to have similar effects, but its addictive potential and safety profiles are not well-established. Another development is the growing popularity of nicotine salt-based large-capacity flavored disposables, a combination of product attributes that consumers appear to value.

In addition to product innovation, manufacturers of illegal e-cigarettes appear to be using marketing innovation to evade regulations. That is, manufacturers discontinue branded products that have received FDA warning letters or other enforcement actions, and relaunch them under a new brand name. This type of activity helps explain the wide swings in the market shares of e-cigarette products that have not received FDA authorization.

Unlike some other illegal markets, illegal e-cigarette markets have not seen rapid adoption of new manufacturing technologies or the use of new technologies in the supply chain. Given the low market share of online retailers, it is reasonable to expect increased growth of online e-cigarette sales, especially to evade regulation.

It has proven difficult for policymakers to develop effective mechanisms to enforce e-cigarette regulations. Like with most technology innovations, there is a lag in legislating laws relating to e-cigarettes; most places have only recently established regulations surrounding e-cigarettes and have not or are just starting to tackle the problem of illegal e-cigarettes. As the law catches up, we might expect enforcement against illegal e-cigarette markets to look more like enforcement against illegal cigarette markets, at least at the individual country level.

An innovative, but controversial, direction to limit illegal cigarette markets and reduce the harms to public health could involve partnerships with cigarette manufacturers. As noted above, despite the involvement of the cigarette industry, track and trace is a promising and scalable approach to limit illegal markets. Even more controversially, major cigarette manufacturers claim to have adopted the long-run strategy of moving away from cigarettes and shift toward e-cigarettes and other lower-risk consumer nicotine products. The cigarette industry, however, has a long history of misleading health claims (70). Nevertheless, cigarette manufacturers may be well-positioned to use sales of e-cigarettes and other lower-risk products to recapture some of the cigarette sales lost when smokers quit. In the United States, the potential of this strategy hinges on the future direction of FDA regulation. Under the FDA’s current regulatory approach, despite the entry of major cigarette manufacturers into the legal e-cigarette market, the illegal e-cigarette market has flourished because it is able to provide popular flavored products.

## 6. Concluding Discussion

In many jurisdictions, the illegal markets for cigarettes and e-cigarettes described in this paper stand alongside legal markets for the same or similar products. The illegal markets arose to evade taxes and regulations, which were adopted as public health measures. In this way, illegal cigarette and e-cigarette markets have important parallels with markets for illegal firearms and for illegally caught fish. Because these illegal markets blunt the ability of public policies to improve social welfare, an obvious response is increased enforcement. Despite long-standing enforcement efforts against illegal cigarette markets, over the past 25 y, illegal markets appear to have had a stable share of about 10 percent of global cigarette consumption. To date, enforcement efforts against the more recent illegal markets for e-cigarettes have been much less effective. A notable example is in the United States, where 86 percent of e-cigarettes consumed are illegal (22).

Paradoxically, like the illegal market for drugs, many of the best measures to address the illegal market for tobacco are implemented alongside the policies that create the illegal markets in the first place. US laws like the Tobacco 21 laws, which increased the minimum legal purchasing age for tobacco products from 18 to 21, decreased smoking rates among 18- to 20-y-olds by 2 to 4 percentage points (71). However, these laws also have the potential to increase the illegal market participation for youths under 21 who continue to consume tobacco products. This duality leaves a difficult challenge to balance illegal markets and public health when crafting tobacco control policies.

Compared to other illegal markets, a surprising and significant difference in the market for illegal cigarettes and e-cigarettes is the limited role of violent organized crime and gangs. In the United States, the diversion of illegal e-cigarettes often comes from the manufacturers themselves, and not a third party, eliminating the need for organized crime involvement. There are instances of violent organized crime or gangs’ involvement in the illegal cigarette and e-cigarette markets; however; no individual group is a key player or is responsible for a significant market share of illegal cigarettes and e-cigarettes. Instead, the supply side of illegal cigarettes and e-cigarettes is dispersed among a variety of individual networks. This may be in part due to the low barriers to entry for small-scale smuggling operations and the concurrent legal markets for cigarettes and e-cigarettes.

However, unlike illegal markets for firearms and illegally caught fish, the existence of illegal e-cigarette markets does not necessarily undermine progress toward the intended policy goal. A growing body of evidence suggests that many consumers see e-cigarettes as substitutes for cigarettes. The US e-cigarette market is in a temporary equilibrium with a highly concentrated legal segment and a more competitive and innovative illegal segment. Although some of the innovations are efforts to avoid regulation, the illegal segment also provides innovative products that adult smokers use to cut down or quit smoking. Successful enforcement efforts against these products could lead to a highly concentrated e-cigarette market with products that consumers find less appealing, which could shift consumer demand back into smoking much riskier combustible cigarettes. Put differently, the existence of illegal e-cigarette markets might be a second-best equilibrium that is better for public health than the alternative of strict enforcement.

## Data Availability

There are no data underlying this work.

## Footnotes

^*^We refer to consumer products that deliver nicotine to distinguish them from pharmaceutical products including nicotine patches and gum. The pharmaceutical products that are most often used to aid in smoking cessation are typically used only for a limited time. While e-cigarettes are also used for smoking cessation, many users consider them as enjoyable products in their own right.

^†^Cigars and loose tobacco are a small share of the legal market and an even smaller share of the illegal market. Heated tobacco products, in which tobacco is heated but not combusted, account for a sizable share in some countries such as Japan.

^‡^Authors’ calculations.

^§^The United Kingdom limits the size of e-cigarette tanks, nicotine strength, and the volume of the refill tanks (56), but has taken the stance that “they’re far less harmful than cigarettes and can help you quit smoking for good” (57).

^¶^A treaty focused addressing tobacco control from a public health approach at a global level. The ITTP extended the treaty to explicitly address illegal tobacco markets and consumption.

^#^To provide some scale for these numbers, the Australian National Drug Strategy Household Survey reports that in 2022-2023, there were 2.3 million smokers and 1.5 million current users of e-cigarettes (58). The Sydney operation’s seizure of 5.6 million cigarettes corresponds to 2.4 cigarettes per current smoker; the seizure of 200,000 e-cigarettes corresponds to 0.13 e-cigarettes per current vaper.

^||^The maximum penalty is $1 million for all violations adjudicated in a single proceeding. Additional penalties may be applied if the violations continue, but which may not exceed $10 million.
